# Selection and Characterization of a Candidate Therapeutic Bacteriophage That Lyses the *Escherichia coli* O104:H4 Strain from the 2011 Outbreak in Germany

**DOI:** 10.1371/journal.pone.0052709

**Published:** 2012-12-21

**Authors:** Maia Merabishvili, Daniel De Vos, Gilbert Verbeken, Andrew M. Kropinski, Dieter Vandenheuvel, Rob Lavigne, Pierre Wattiau, Jan Mast, Catherine Ragimbeau, Joel Mossong, Jacques Scheres, Nina Chanishvili, Mario Vaneechoutte, Jean-Paul Pirnay

**Affiliations:** 1 Laboratory for Molecular and Cellular Technology, Queen Astrid Military Hospital, Brussels, Belgium; 2 Eliava Institute of Bacteriophage, Microbiology and Virology, Tbilisi, Georgia; 3 Laboratory of Bacteriology Research, Ghent University, Ghent, Belgium; 4 Laboratory for Foodborne Zoonoses, Public Health Agency of Canada, Ontario, Canada; 5 Department of Molecular and Cellular Biology, University of Guelph, Guelph, Ontario, Canada; 6 Laboratory of Gene Technology, KU Leuven, Heverlee, Belgium; 7 Unit of Highly Pathogenic & Foodborne Zoonoses, Veterinary and Agrochemical Research Centre, Brussels, Belgium; 8 Electron Microscopy Unit, Veterinary and Agrochemical 8 Research Centre, Brussels, Belgium; 9 Surveillance and Epidemiology of Infectious Diseases, Laboratoire National de Santé, Luxembourg, Luxembourg; 10 Maastricht University Medical Centre, Maastricht, The Netherlands; 11 European Centre for Disease Prevention and Control, Stockholm, Sweden; University of California Merced, United States of America

## Abstract

In 2011, a novel strain of O104:H4 *Escherichia coli* caused a serious outbreak of foodborne hemolytic uremic syndrome and bloody diarrhea in Germany. Antibiotics were of questionable use and 54 deaths occurred. Candidate therapeutic bacteriophages that efficiently lyse the *E. coli* O104:H4 outbreak strain could be selected rather easily from a phage bank or isolated from the environment. It is argued that phage therapy should be more considered as a potential armament against the growing threat of (resistant) bacterial infections.

## Introduction

From May to July 2011, Germany was struck by the largest outbreak of hemolytic uremic syndrome (HUS) and bloody diarrhea caused by *Escherichia coli* ever reported [1]. A total of 3,816 cases were reported, 845 (22%) of which involved HUS. This high rate of HUS was the first indicator that the bacterial cause of illness was not a typical enterohaemorrhagic *E. coli* (EHEC) strain [2]. It was shown to be a highly pathogenic enteroaggregative *E. coli* (EAEC) strain, which in addition carried the EHEC genes for the classical HUS-associated Shiga toxin 2 [3]–[4]. Notwithstanding the timeliness of the German surveillance system (reporting occurred faster than required by law) for HUS and Shiga toxin-producing *E. coli* notifiable diseases [5], no less than 54 patients died. Eventually, sprouts were identified as the most likely outbreak vehicle [6]. The outbreak strain was resistant to beta-lactam antibiotics and third-generation cephalosporins and was partially resistant to fluoroquinolones, but sensitive to carbapenems and ciprofloxacin [1]. However, a Cochrane database review analyzing seven randomized controlled clinical trials of pediatric HUS associated with EHEC infections found no benefit of antibiotic treatment over supportive therapy alone [7]. Moreover, the use of antibiotics to treat Shiga toxin producing *E. coli* infections has been discouraged because it has been shown to release toxins. The genes for the Shiga toxin are actually not bacterial genes, but (bacterio)phage genes. When an *E. coli* bacterium gets infected with a temperate phage harboring a Shiga toxin gene, upon integration of the phage genome (as prophage) into the bacterial genome (lysogeny), it can be expressed by the bacterium, which then becomes pathogenic. Some types of antibiotics have been shown to induce the so-called SOS response in bacteria, i.e. a ubiquitous response to DNA damage, which induces phage replication and lytic cycle [8]. As such, the use of antibiotics may be helping Shiga toxin genes to spread. Phage-mediated transfer of bacterial virulence, fitness and antibiotic resistance is a negative (from the human point of view) consequence of bacterial phage coevolution.

Paradoxically, phages from a very different, constitutionally lytic genus could also help fight EHEC and EAEC. Phages do play a major role in controlling bacterial densities in the biosphere, including humans, which is the basis of sustainable phage therapy [9]. For example, phages appear to be key players in ending cholera epidemics. Faruque *et al*. [10] observed that seasonal epidemics of cholera inversely correlated with the prevalence of environmental cholera phages. Phages could be used therapeutically, as an additional tool or in combination with antibiotics, to treat bacterial infections that do not respond to conventional antibiotics. Importantly, they can be chosen to be harmless to the commensal bacteria, such as those of the gut microflora. The oral application of phages to humans is likely to be very safe [11] and several phage based preparations were given regulatory approval and are commercially available for the decontamination of foods [12]. Muniesa *et al.* [13], in a review on experimental treatment and prevention options for HUS, reported phages to be an effective tool for disinfection of various vegetables and meat products contaminated by *E. coli* strain O157. Steers that received phage by the rectal route showed a 100-fold fecal titer decrease of O157 compared to controls. In sheep, oral T4-like coliphage reduced the O157 counts in the caecum and rectum. Recently, French scientists isolated three morphologically different types of tailed phages that infect *E. coli* O104:H4. The cocktail of all three phages showed sustained replication in the intestines of mice, but without decreasing the intestinal titer of O104:H4 cells [14].

Here we present the results of an *ad hoc* multidisciplinary working group, including institutions involved in infectious diseases surveillance, phage therapy and molecular biology research, which set out to isolate, select and characterize candidate therapeutic phages active against the *E. coli* O104:H4 outbreak strain.

## Materials and Methods

Isolation and selection of *E. coli* O104:H4 phages were performed according to the methods described by Merabishvili *et al*. [15]. Considering laboratory safety in the handling of EHEC, all manipulations involving the O104:H4 outbreak strain were performed in a dedicated biosafetylevel 3 facility. A set of 16 phages known to infect *E. coli* was screened against the outbreak strain. The set included the well-known T4 phage, nine phages from the therapeutic phage library of the Eliava Institute of Bacteriophage, Microbiology and Virology in Tbilisi (Georgia) and six phages that were recently isolated from wastewater of the Queen Astrid Military Hospital in Brussels.

The lytic potential of the 16 phages was tested on a collection of 92 *E. coli* strains using the horizontal bacterial strips method on agar plates [15]. The tested collection included ten different pathogenic *E. coli* serotypes (Table 1) and 82 clinical and non-clinical *E. coli* strains.

**Table 1:** Susceptibility of ten pathogenic *E. coli* serotypes to infection with phage GEC-3S.

	Serotype									
	O26:K60	O55:K59	O86:K61	O104:H4	O111:K58	O111:K60	O125:K70	O128:K67	O142:K86	O157:H7
**GEC-3S**	−	−	−	cl	−	−	cl	−	–	–

−, no lysis; cl, confluent lysis.

Phage genome sequence was determined by pyrosequencing (454 technology) according to Kropinski *et al*. [16] and analyzed according to Vandersteegen *et al*. [17]. Open reading frames were predicted, and their functions defined, by ORF finder (http://www.ncbi.nlm.nih.gov/projects/gorf/), GeneMark™ [18] and BLASTp [19] programs, followed by manual confirmation. EMBOSS stretcher [20], [21] was used to compare DNA homology between phages.

## Results and Discussion

**Figure 1:**
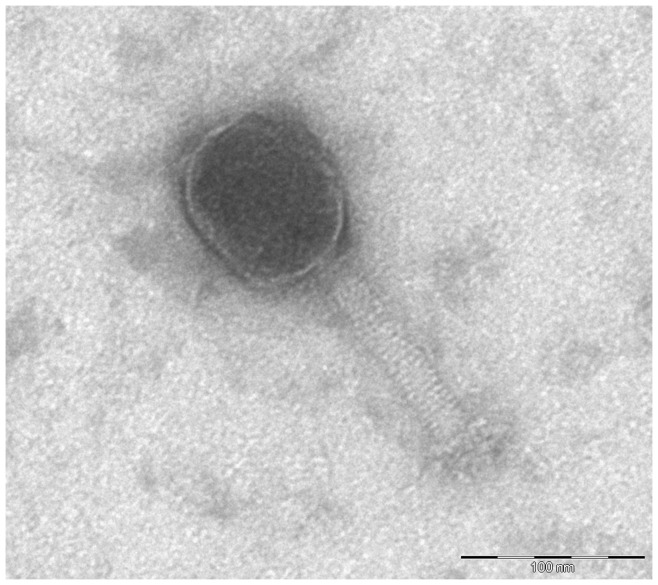
Transmission electron micrographs of myovirus GEC-3S. Phage particles were analyzed by transmission electron microscopy as described by Imberechts *et al*. [27]. The head is somewhat elongated, appears hexagonal in outline and has a mean diameter of 102 nm (SEM = 3 nm). The head is separated from the tail by a neck. The tail is a rigid, contractile tube with a mean length of 109 nm (SEM = 1 nm). It appears cross-banded, suggesting helical symmetry. The tail ends in short and long fibers. The latter do not show up very well on this micrograph.

**Figure 2:**
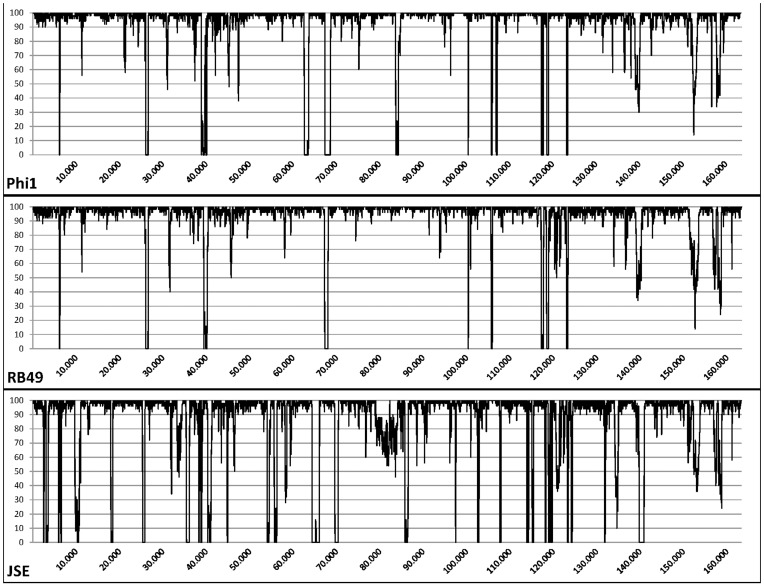
Pairwise DNA homology of GEC-3S and phi1, GEC-3S and RB49, GEC-3S and JSE, compared using a sliding window of 50 bp.

Only one out of 16 phages, myovirus GEC-3S (Fig. 1) from the Eliava collection, showed activity against the outbreak strain. In addition, two myoviruses, QAMH-FFP.1 and QAMH-NES.1, were newly isolated from the wastewater of the Brussels Military Hospital using the culture enrichment method with the original outbreak strain as the host. In contrast to phages QAMH-FFP.1 and QAMH-NES.1, phage GEC-3S could be propagated in *E. coli* strain K12 and this without significant decrease of titer or activity against strain O104:H4, which is not negligible in the context of an eventual production of therapeutic or prophylactic phage preparations. The non-pathogenic K12 strain is commonly used in Good Manufacturing Practices (GMP) and clean-room compliant preparation of medicinal products, whereas the handling of the O104:H4 strain requires strict biocontainment precautions. Phages QAMH-FFP.1 and QAMH-NES.1 showed no activity against the K12 strain. Of the ten tested pathogenic *E. coli* serotypes, only two – O104:H4 and O125:K70– were susceptible to phage GEC-3S (Table 1). The overall activity of the phage against 92 pathogenic and non-pathogenic *E. coli* strains proved to be 20.7% (19/92). Complete genome sequencing (Accession number HE978309) revealed that phage GEC-3S belongs to the genus of T4-like viruses with high similarity to the representatives of the RB49 group. Two hundred and seventy-six open reading frames were predicted and their functions were defined. At the nucleotide level, GEC-3S showed high homology to phi1, RB49 and JSE phages, respectively 94.6, 93.6 and 88.0%. The shared differences between GEC-3S and the other phages are spread throughout the whole genome and in particular in the regions between 39,000–40,000, 67,000–68,000, 100,000–110,000 and 115,000–125,000 bp, loci which mostly comprise ORFs coding for small hypothetical proteins and homing endonucleases (Fig. 2). Phage GEC-3S harbors six unique genes that are not present in the genomes of the other representatives of the RB49 group (phi1p263, RB49p263, EpJSE_00266) (Table 2). Four of these genes are ORFans, while the other two are coding homing endonucleases, extremely widespread enzymes in T-even-like phages according to available genomic data [22]. The only gene that is typical for the other three phages from this group, but is not present in GEC-3S is a hypothetical protein of 51 amino acids length (Table S1).

**Table 2:** ORFs of phage GEC-3S not presented in any other representative of the RB49 group.

N	Amino acid length	E value	Putative functions/Known Homologs*
ORF 63	251	5×10^−16^	putative homing endonuclease RB16 6 (Enterobacteria phage RB16)
ORF 137	246	1×10^−9^	putative H-N-H-endonuclease P-TflX (Enterobacteria phage T5)
ORF 163	32	–	ORFan
ORF 171	44	–	ORFan
ORF 265	80	–	ORFan
ORF 266	66	–	ORFan

* Putative functions and known homologs of ORFs were identified using BlastP against the non-redundant database.

Being a T4-like phage (of the RB49 group), much is known about the properties and safety of phage GEC-3S, further strengthening its appropriateness for therapeutic applications. In general, temperate phages are considered inappropriate for use as therapeutic phages because they can transduce unwanted genes. Sequence similarity searches against the non redundant NCBI database confirmed the lytic (non temperate) character of phage GEC-3S and the absence of genes known to encode virulence or pathogenicity associated, or potentially allergenic, proteins. This is important, as the *E. coli* outbreak strain itself is a good illustration of phage-mediated increased pathogenicity.

Because sequencing of the O104:H4 outbreak strain failed to provide clues about its origin and evolution, it is unclear whether we may expect similar upcoming outbreaks to occur recurrently or spontaneously in the future [23]. Even though the epidemic has been declared finished, the health community should explore new (prophylactic) therapies. Theoretically, phage GEC-3S could have been used to help control (in humans, animals, the environment or food) the O104:H4 outbreak that caused the death of more than 50 patients. A cocktail of purified and fully defined and safe phages selected against the most problematic EHEC and EAEC strains could be produced and stored as an additional antimicrobial when an outbreak similar to the one witnessed in Germany occurs in the future. The high bacterial strain specificity of phages make it necessary to provide cocktails for treatment of disease. If required, new phages could be added to the cocktail. The isolation and selection process of the here presented phages took only 3 days to complete. A useful strategy would be to set up an international bank by isolating a large number of phages from E. coli-pathogen-rich sites using strain K12, checking their ability to grow efficiently on all the important pathogenic E. coli strains available (e.g. O104:H4), and from these choosing a set that provides broad coverage of the known problem strains. It would be an advantage if these were T4-related phages, about which so much is known. Ideally, component phage identification and detailed characterization and maintenance of this bank should be supported by WHO and/or the European and American CDCs [24]. The O104:H4 outbreak caused considerable suffering and resulted in a strain on healthcare and public health systems [25], which could provide an incentive for competent authorities and physicians to use phages, as additional tools, in the prevention and treatment of otherwise virtually untreatable infections. Meanwhile, preventive microbiology remains crucial for early detection of major health threats caused by infectious diseases [26].

## Supporting Information

Table S1 **Comparison of ORFs/genes of phage GEC-3S with other representatives of the RB49 group.**
(XLSX)
